# Machine learning model for predicting severe infection in children with idiopathic nephrotic syndrome: multicenter retrospective study

**DOI:** 10.1186/s13052-025-02149-7

**Published:** 2025-11-25

**Authors:** Sijie Yu, Wenhao Tang, De Zhang, Fuwei Shen, Anshuo Wang, Han Chen, Hongxing Chen, Fanghong Zhang, Li Xiao, Yan Li, Zongwen Chen, Li Wang, Mo Wang, Haiping Yang, Qiu Li

**Affiliations:** 1 Department of Nephrology, Children’s Hospital of Chongqing Medical University, National Clinical Research Center for Child Health and Disorders, Ministry of Education Key Laboratory of Child Development and Disorders, Chongqing Key Laboratory of Pediatric Metabolism and Inflammatory Diseases, 136 Zhongshan 2nd Road, Yuzhong District, Chongqing, China; 2 National Center for Applied Mathematics, Chongqing Normal University, Chongqing, China; 3 Big Data Center for Children’s Medical Care, Children’s Hospital of Chongqing Medical University, Chongqing, China; 4 Department of Nephrology, Xuzhou Children’s Hospital, Xuzhou, Jiangsu Province China; 5 Department of Pediatrics, Chongqing University Three Gorges Hospital, Chongqing, China; 6 Department of Nephrology, Chengdu Women’s and Children’s Central Hospital, Chengdu, Sichuan Province China

**Keywords:** Idiopathic nephrotic syndrome, Severe infection, Children, Machine learning, Prediction model

## Abstract

**Background:**

Infection is a common complication of idiopathic nephrotic syndrome (INS), and early identification of severe infection can improve patient outcome.

**Methods:**

This multicenter retrospective study developed and validated machine learning (ML) models that predict severe infection in children with INS. The derivation cohort (*n* = 2357) consisted of INS patients at one institution, and was separated into a training set and testing set. The external validation set (*n* = 372) consisted of INS patients from three other hospitals. Data were collected for 41 variables, and ten of them were then selected by univariate analysis and Least Absolute Shrinkage and Selection Operator (LASSO) regression. Ten ML models were compared, and the best one was identified using receiver operating characteristic (ROC) analysis and other methods.

**Results:**

The incidence rate of severe infection was 6.8% in the derivation cohort. The Light Gradient Boosting Machine (LightGBM) model had the best predictive performance (accuracy: 0.843, precision: 0.843, recall: 0.842, F1: 0.843, sensitivity: 0.842, specificity: 0.844, AUROC:0.912, AUPRC:0.915). The ten predictors were C-reactive protein, hemoglobin, white blood cells, activated partial thromboplastin time, creatinine, high-density lipoprotein, corrected serum calcium, complement 3, and number of immunosuppressants, and incidence of SRNS. This model had an AUROC of 0.979 and AUPRC of 0.842 in the external validation cohort.

**Conclusion:**

A LightGBM model for predicting severe infection in patients with INS had excellent performance. Future applications of this model may provide an effective, convenient, and cost-effective approach for early identification of severe infection in children with INS.

**Supplementary Information:**

The online version contains supplementary material available at 10.1186/s13052-025-02149-7.

## Background

Idiopathic nephrotic syndrome (INS) is the most common glomerular disease in children, has an annual incidence of 1.4 to 6.1 per 100,000 children depending on ethnicity, and has a high incidence in Southeast Asia and East Asia [1, 2]. The 2022 clinical practice recommendations of the International Pediatric Nephrology Association (IPNA) consider nephrotic-range proteinuria, hypoalbuminemia, and edema to be the three major signs of INS [3]. Infection is the leading cause of INS recurrence and hospitalization, and can lead to severe infection, morbidities, and mortality [4–6].

INS patients have increased urinary excretion of IgG and complement opsonins [7] and their treatment with glucocorticoids and immunosuppressants [2, 8] leads to weakened immunity and increased risk of infection [9]. Previous studies reported the incidence of infection in children with INS ranged from 8 to 84%, depending on diagnostic criteria, treatment regimen, and duration of follow-up [10–13]. Most INS patients with infections have atypical presentation, and delayed diagnosis by non-nephrologists may delay the implementation of necessary interventions that could eliminate the infection or slow its progression. Consequently, accurate and timely diagnosis of INS that is complicated by severe infection is crucial for improving patient outcome and decreasing the economic burden of hospitalization. Although some studies have identified risk factors associated with severe infection in pediatric patients with INS by use of traditional Cox regression and binary logistic regression, the accuracy and generalizability of these results are limited by the small sample sizes and lack of external validation [4, 14, 15].

The development of electronic medical record (EMR) systems has greatly facilitated the collection of clinical data, and the resulting analyses are therefore less laborious, more accurate, and more reliable. The recent development of artificial intelligence (AI) models based on machine learning (ML) algorithms has led to the clinical use of these models with data from EMR systems. The clinical application of AI models is now a very active field that is undergoing rapid development [16, 17]. For example, researchers recently found that models using ML algorithms were able to predict severe infection with high accuracy [18–21]. The success of these and other ML models was achieved by the use of training on datasets and the application of mathematical functions or rules, a method that provides classification and predictive outputs with high precision [22]. Traditional Cox regression models and binary logistic regression models rely on linear assumptions, but ML algorithms incorporate linear and non-linear assumptions, and this can facilitate the identification of new features and improve the accuracy of predictions. In the present study of cohorts from multiple centers, we constructed and validated explainable AI models for the early and accurate prediction of severe infection in pediatric patients with INS.

## Methods

### Study population

This retrospective study initially examined children diagnosed with INS at Chongqing Medical University Children’s Hospital (CMUCH) from January 2012 to December 2022. This cohort was the derivation dataset. All children who were 1 month to 18-years-old and met the IPNA criteria (see below) for INS were considered for inclusion. Children were excluded if they had congenital NS, secondary NS (IgA nephropathy, lupus nephritis, purpuric nephritis, etc.), an initial diagnosis of INS with severe infection, or were missing significant laboratory data.

### Data processing and selection of variables

The selection of variables for construction of the prediction models was based on clinical experience and review of relevant publications that examined children with INS and severe infection. The data included demographic characteristics, laboratory results, and medication history, and were recorded upon admission and retrieved from the EMR system. Because an excess of missing data could affect the accuracy of predictions, any variable that was missing in more than 30% of the patients was excluded (Supplementary Figure S1). The following 41 variables were ultimately included: duration of steroid therapy, body weight, age, sex, white blood cell count (WBC), the percentage of lymphocytes (LYM%), the percentage of neutrophils (NEU%), absolute monocyte count (AMC), hemoglobin (Hb), platelet count (PLT), C-reactive protein (CRP), total protein (TP), albumin (ALB), globulin (GLB), alanine aminotransferase (ALT), glutamyl transpeptidase (GGT), creatinine, urine protein, urine red blood cell count (uRBC), 24 h urine protein, cystatin C, triglycerides (TGs), low-density lipoprotein cholesterol (LDL), high-density lipoprotein cholesterol (HDL), prealbumin, lactate dehydrogenase (LDH), serum magnesium (Mg), corrected serum calcium, fibrinogen, activated partial thromboplastin time (APTT), thrombin time (TT), D-dimer, immunoglobulin G (IgG), immunoglobulin E (IgE), immunoglobulin A (IgA), immunoglobulin M (IgM), complement 3 (C3), complement 4 (C4), type of immunosuppressant therapy, recurrence, SRNS.

In addition, methods to decrease the impact of outliers and one-hot encoding of categorical variables were performed. To mitigate the impact of data gaps, multiple imputation methods were used, an approach usually recommended for handling missing data during model development [23, 24]. Normalization methods were also used to eliminate the impact of dimensional differences among variables, because these differences could affect model performance.

To identify the variables most relevant to severe infection, a two-step approach was used. Firstly, a univariate analysis of the derivation dataset showed that 30 variables were significantly different between the cohorts with and without severe infection. Then, these 30 variables were utilized in Least Absolute Shrinkage and Selection Operator (LASSO) regression to select the most appropriate clinical variables for constructing the models. This approach can improve the predictive accuracy of ML models while preventing overfitting [25].

### Definitions of INS and severe infection

The diagnosis of INS was according to the three standard criteria of the IPNA: urinary protein-creatinine ratio (UPCR) ≥ 200 mg/mmol (2 mg/mg) in a spot urine test, or proteinuria ≥ 1000 mg/m^2^ in a 24-h urine sample, or a urine dipstick result of (3 + (300–1000 mg/dL) or 4 + (≥ 1000 mg/dL); and hypoalbuminemia (serum albumin < 30 g/L) or edema when serum albumin is not available [3].

The criteria for relapse of nephrotic syndrome were: urine dipstick ≥ 3 + (≥ 300 mg/dL) or UPCR ≥ 200 mg/mmol (≥ 2 mg/mg) on a spot urine sample on 3 consecutive days, with or without reappearance of edema in a child who had previously achieved complete remission. The definition of steroid-sensitive nephrotic syndrome (SSNS) was complete remission within 4 weeks of PDN at standard dose and the definition of steroid-resistant nephrotic syndrome (SRNS) was lack of complete remission within 4 weeks of treatment with PDN at standard dose [3].

Infection was diagnosed based on clinical symptoms, laboratory results, imaging, and microbiology test results. Severe infection in children [26] and adults [27] were according to established guidelines. In particular, severe infection was diagnosed when a patient had an infection that required fluid resuscitation and medication to maintain blood pressure; or required mechanical ventilation; or required admission to an intensive care unit due to acute respiratory distress syndrome (ARDS), multiple organ dysfunction syndrome (MODS), or septic shock. Additionally, patients without a clear diagnosis of infection who presented with signs and symptoms of inflammation and had the following three criteria were also considered to have severe infection: changes in general signs (at least two); changes in inflammatory markers (at least one); and at least one change in organ function, tissue perfusion, or hemodynamic status, as described in our previous study [4] (Supplementary Table S1).

### Development and comparison of different models

A cohort of patients from CMUCH who were hospitalized from January 2012 to December 2022 was used to establish the training set (70%) and testing set (30%) for development of the predictive models. Datasets from the three other hospitals were utilized for external validation (see below).

The ten best clinical variables selected by LASSO regression were utilized to construct ten predictive models: two linear models, three non-linear models, and five models ensemble learning models. The ten specific ML models were: Logistic Regression (LR), Random Forest (RF), K-Nearest Neighbors (KNN), Naïve Bayes (NB), Support Vector Machine (SVM), Extreme Gradient Boosting (XGBoost), Adaptive Boosting (AdaBoost), Light Gradient Boosting Machine (LightGBM), Decision Tree (DT), and Gradient Boosting Machine (GBM). The Synthetic Minority Over-sampling Technique (SMOTE) was utilized to account for sample imbalance [28]. In addition, to ensure the reliability and generalizability of the models, five cross-validations and GridSearch were used to select the optimal hyperparameters.

The performance of each model was assessed by calculation of multiple indexes: area under the receiver operating characteristic curve (AUROC), area under the precision-recall curve (AUPRC), accuracy, precision, recall, F1 score, sensitivity, and specificity [29].

### External validation

The external validation cohort consisted of children admitted to CUTGH, XZCH, or CWCCH from 2023 to 2024, and the inclusion and exclusion criteria were identical to those of the derivation cohort.

### Interpretation of models

A ML model is considered to be a “black box”, making it difficult to interpret the results. The SHapley Additive exPlanations (SHAP) method, which is derived from coalitional game theory, calculates a value for each variable, determines its contribution to the prediction results, and provides interpretations for local and global predictions [30, 31].

The global predictions provide attribution values with consistency and accuracy for each variable in a model, and can show the associations between input variables and severe infection. The local predictions provide a specific prediction for an individual child based on relevant data [30].

### Statistical analysis

Patients with INS and infection were categorized as having severe infection or non-severe infection. Continuous variables that had a normal distribution were presented as mean ± standard deviation and compared using Student’s *t*-test. Continuous variables that had non-normal distributions were presented as median and interquartile and compared using the Mann–Whitney U test or the Kruskal–Wallis H test. Categorical variables were presented as frequency and percentage and were compared using the Chi-square test or Fisher’s exact test. A two-tailed P value below 0.05 was considered statistically significant. All analysis, computations, and mapping were performed using Python version 3.9, R version 4.4.1, and SPSS version 22.0.

### Ethics statement

This study was reviewed and approved by the Ethics Committee of CMUCH (approval number: 2024-KY-0212), Xuzhou Children's Hospital (XZCH, approval number: 2024–05–57-H57), Chongqing University Three Gorges Hospital (CUTGH, approval number: No.2025(010)), and Chengdu Women's and Children's Hospital (CWCCH, 2024–65), and also adhered to ethical principles of the World Medical Association Declaration of Helsinki. This study was a retrospective analysis that used anonymized patient data; the authors have no potential commercial interests in the outcome; and the research plan was developed after the 10 year period of data collection. Because of the large sample size and long period of data collection, it was not possible to contact all parents for informed consent regarding the use of the anonymized retrospective data.

## Results

### Baseline characteristics of the derivation cohort

**Fig. 1:**
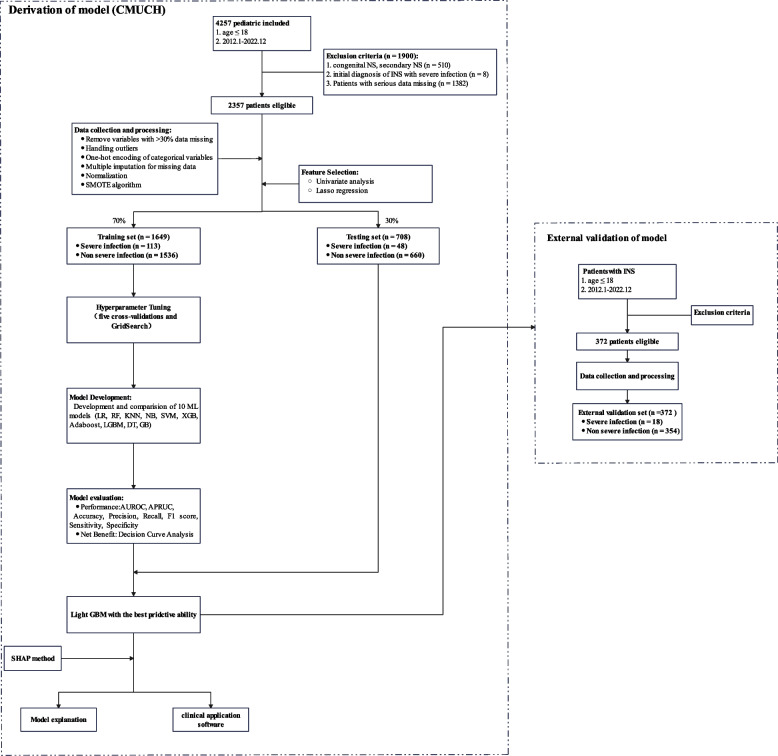
Study design. LR: logistic regression; RF: random forest, KNN: K-nearest neighbors; NB: naïve Bayes; SVM: support vector machine; XGBoost: extreme gradient boosting; AdaBoost: adaptive boosting; LGBM: light gradient boosting machine; DT: decision tree; GBM: gradient boosting machine

We initially performed a retrospective examination of the records of 4257 children from CMUCH who had NS (Fig. 1). After application of the inclusion and exclusion criteria, there were 2357 patients (161 [6.8%] with severe infection) in the derivation cohort. The mortality rate from severe infection was 13/161 (8.1%). We used computerized randomization to divide these patients into a training set of 1649 patients (70%; 113 [6.85%] with severe infection) and a testing set of 708 patients (30%; 48 [6.77%] with severe infection). The external validation set had 372 patients (18 [4.84%] with severe infection). The baseline data of the training set, testing set, and external validation set are provided in Supplementary Table S2.

Analysis of the 41 baseline variables in all 2357 eligible patients in the derivation cohort indicated that patients with non-severe infection and severe infection had significant differences in 30 variables (Table 1). In particular, the group with non-severe infection had greater values for body weight, age of onset, Hb, ALB, GLB, HDL, Mg,fibrinogen, TT, IgG, IgA, C3, and duration of steroid use. The group with severe infection had greater values for WBC, AMC, PLT, CRP, ALT, GGT, creatinine, uRBCs, 24 h urine protein, cystatin C, LDH, corrected serum calcium, APTT, D-dimer, and IgE, used more types of immunosuppressants, and had a higher incidence of SRNS.

**Table 1:** Baseline characteristics of INS patients with non-severe infections and severe infections in the entire derivation cohort^†^

Characteristic	Non-Severe^*^	Severe^*^	*P*
	***N* = 2196**	***N* = 161**	
Weight, kg	**25.00 [18.00, 36.00]**	21.50 [15.50, 32.00]	0.001
Age at onset, months	**2662.50 [1730.00, 3893.75]**	2097.00 [1349.00, 3779.00]	0.001
Gender			0.558
Female	621 (28.28%)	49 (30.43%)	
Male	1575 (71.72%)	112 (69.57%)	
WBC × 10^9^/L	10.15 [7.76, 13.53]	**15.55 [10.83, 21.30]**	< 0.001
LYM, %	34% [23%, 45%]	34% [22%, 47%]	0.559
NEU, %	61% [48%, 73%]	59% [45%, 73%]	0.149
AMC × 10^9^/L	0.34 [0.21, 0.52]	**0.63 [0.37, 0.93]**	< 0.001
Hb, g/L	**135.00 [126.00, 145.00]**	108.00 [91.00, 125.00]	< 0.001
PLT × 10^9^/L	376.50 [304.00, 469.00]	**449.00 [332.00, 577.00]**	< 0.001
CRP, mg/L	8.00 [8.00, 8.00]	**8.00 [8.00, 24.00]**	< 0.001
TP, g/L	49.10 [41.10, 58.40]	52.00 [40.70, 60.80]	0.120
ALB, g/L	**23.45 [17.20, 36.20]**	17.80 [14.50, 27.30]	< 0.001
GLB, g/L	**20.40 [18.20, 23.00]**	18.30 [15.90, 21.60]	< 0.001
ALT, U/L	15.60 [11.30, 23.10]	**26.00 [18.00, 42.00]**	< 0.001
GGT, U/L	15.00 [10.00, 24.20]	**27.00 [13.70, 52.00]**	< 0.001
Creatinine, µmol/L	38.00 [30.00, 50.00]	**48.00 [34.60, 89.50]**	< 0.001
Urine protein			0.096
No	514 (23.41%)	47 (29.19%)	
Yes	1682 (76.59%)	114 (70.81%)	
uRBC/HPF	4.00 [1.00, 14.00]	**10.00 [2.00, 48.00]**	< 0.001
24 h urine protein, mg	78.79 [16.96, 159.84]	**118.86 [13.50, 269.45]**	< 0.001
Cystatin C, mg/L	0.93 [0.78, 1.13]	**1.16 [0.90, 1.77]**	< 0.001
TG, mmol/L	2.30 [1.45, 3.55]	2.53 [1.62, 4.05]	0.064
LDL, mmol/L	4.51 [2.91, 7.34]	5.27 [2.81, 7.98]	0.337
HDL, mmol/L	**2.05 [1.58, 2.63]**	1.42 [1.00, 2.05]	< 0.001
Prealbumin, mg/L	210.00 [150.00, 280.00]	210.00 [139.00, 330.00]	0.279
LDH, U/L	265.00 [226.00, 313.00]	**356.00 [275.00, 541.00]**	< 0.001
Mg, mmol/L	**0.79 [0.71, 0.87]**	0.71 [0.59, 0.80]	< 0.001
Corrected serum calcium	3.35 [2.59, 3.72]	**3.56 [3.07, 3.80]**	< 0.001
Fibrinogen, g/L	**4.25 [3.02, 5.56]**	5.59 [3.80, 8.06]	< 0.001
APTT, s	28.10 [25.00, 32.00]	**34.50 [28.80, 45.20]**	< 0.001
TT, s	**17.40 [16.60, 18.50]**	17.20 [15.90, 18.20]	0.008
D-dimer, µg/mL	0.53 [0.23, 1.39]	**2.27 [0.79, 8.50]**	< 0.001
IgG, g/L	**4.23 [2.55, 6.55]**	2.96 [1.35, 4.98]	< 0.001
IgE, g/L	107.00 [30.78, 416.25]	**165.00 [33.60, 624.00]**	0.031
IgA, g/L	**1.24 [0.86, 1.80]**	1.01 [0.71, 1.43]	< 0.001
IgM, g/L	1.65 [1.17, 2.29]	1.51 [1.11, 2.09]	0.115
C3, g/L	**1.00 [0.86, 1.16]**	0.89 [0.68, 1.08]	< 0.001
C4, g/L	0.21 [0.17, 0.26]	0.22 [0.16, 0.28]	0.517
Corticosteroid usage, days	**592.50 [191.75, 1264.00]**	314.00 [100.00, 822.00]	< 0.001
Immunosuppressant usage			< 0.001
None	**440 (20.04%)**	17 (10.56%)	
1 type	**852 (38.80%)**	46 (28.57%)	
2 types	709 (32.29%)	**65 (40.37%)**	
3 types	185 (8.42%)	**29 (18.01%)**	
4 types	10 (0.46%)	**4 (2.48%)**	
Recurrence			0.340
No	630 (28.69%)	40 (24.84%)	
Yes	1566 (71.31%)	121 (75.16%)	
SRNS			0.027
No	**1540 (70.13%)**	99 (61.49%)	
Yes	656 (29.87%)	**62 (38.51%)**	

*WBC* White blood cell count, *LYC* Lymphocyte, *NEU* Neutrophil, *AMC* Absolute monocyte count, *Hb* Hemoglobin, *PLT* Platelets, *CRP* C-reaction protein, *TP* Total protein, *ALB* Albumin, *GLB* Globulin, *ALT* Alanine aminotransferase, *GGT* Glutamyl transpeptidase, *uRBC* urine red blood cell count, *LDL* Low-density lipoprotein, *HDL* High-density lipoprotein, *LDH* Lactate dehydrogenase, *Mg* serum magnesium, *Total Ca* Total serum calcium, *APTT* Activated partial thromboplastin time, *TT* Thrombin time, *IgG* Immunoglobulin G, *IgE* Immunoglobulin E, *IgA* Immunoglobulin A, *IgM* Immunoglobulin M, *C3* Complement 3, *C4* Complement 4, *SRNS* Steroid-resistant nephrotic syndrome

^†^Each value indicates median [IQR] or N (%)

^*^Bold numbers indicate significantly higher values

### Selection of model variables

**Fig. 2:**
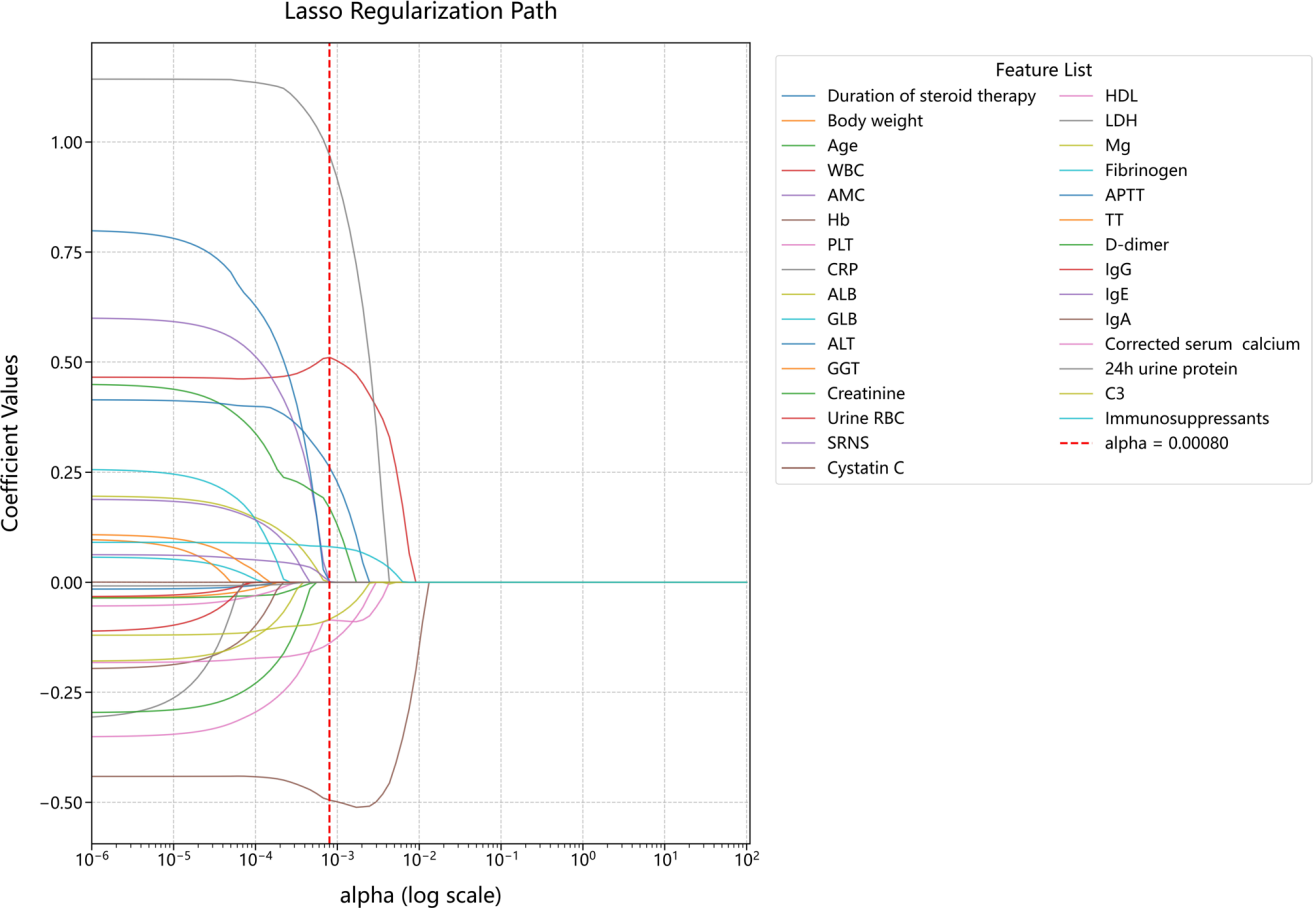
Screening for optimal variables based on LASSO regression analysis in the entire derivation cohort. WBC: white blood cell count; AMC: absolute monocyte count; Hb: hemoglobin; PLT: platelets; CRP: C-reaction protein; TP: total protein; ALB: albumin; GLB: globulin; ALT: alanine aminotransferase; GGT: glutamyl transpeptidase; uRBC: urine red blood cell count; HDL: high-density lipoprotein; LDH: lactate dehydrogenase; Mg: serum magnesium; APTT: activated partial thromboplastin time; TT: thrombin time; IgG: immunoglobulin G; IgE: immunoglobulin E; IgA: Immunoglobulin A; C3: complement 3; C4: complement 4; SRNS: steroid-resistant nephrotic syndrome

We utilized univariate analysis and LASSO regression to select variables to be used in the ten different ML models (Fig. 2). This analysis adjusted the regularization coefficient (lambda) to prevent over-fitting; all models also included number of immunosuppressants used, based on previous research [29]. This analysis led to the identification of ten significant variables (best predictors) for inclusion in the ML models. The associated shrinkage parameter (lambda.1se) was 0.0080. These ten variables were CRP, Hb, WBC, APTT, creatinine, HDL, C3, corrected serum calcium, number of immunosuppressants used, and incidence of SRNS.

### Development and comparison of prediction models

**Fig. 3:**
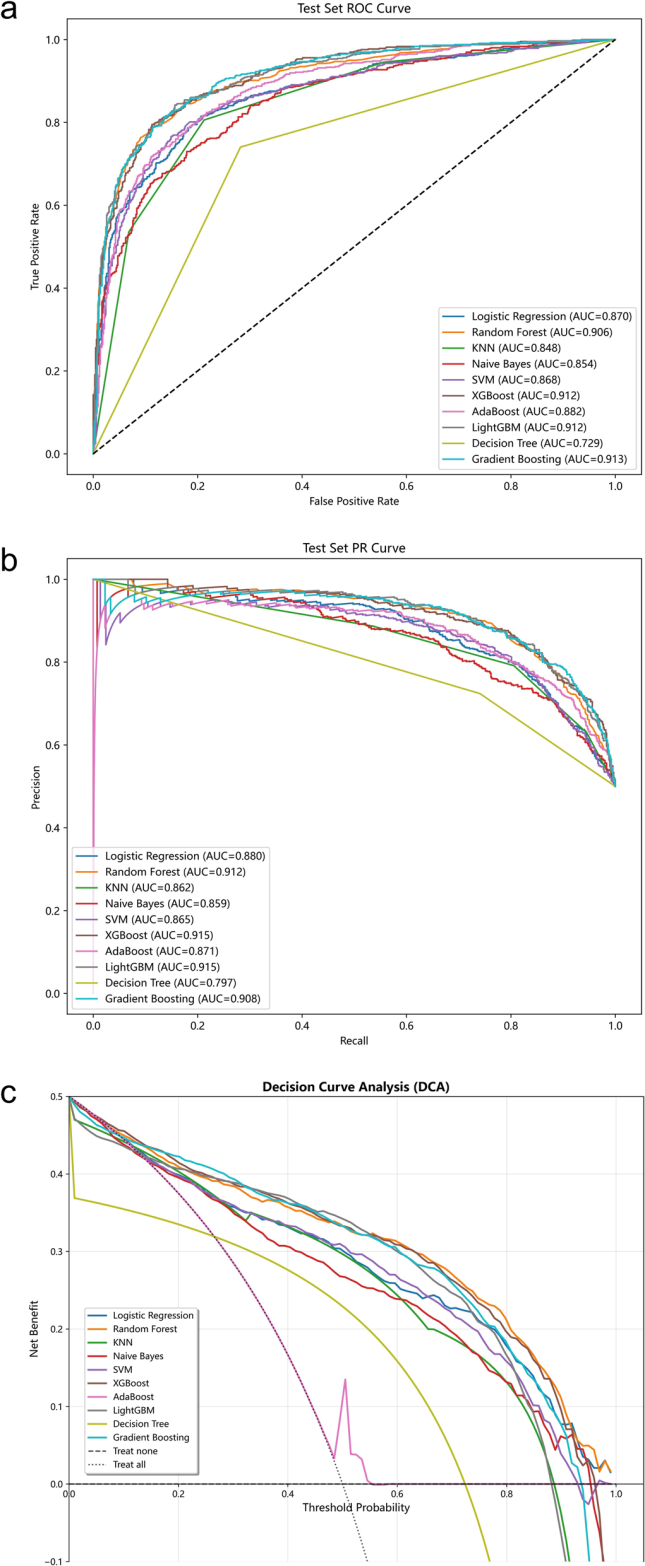
Performance of the ten ML models in prediction of severe infection in the testing set based on AUROC **a**, AUPRC, **b** and DCA **c**

We then used these ten variables to generate ten different ML models to predict severe infection in children with INS. After tuning the hyperparameters, each ML model was trained using the training set (n = 1649) and model performance was evaluated using the testing set (n = 708). We then used AUROC curves and AUPRC curves to assess the performance of each model in the testing set (Fig. 3a, b), and calculated the accuracy, precision, recall, F1 score, sensitivity, and specificity of each model (Table 2) based on the selected hyperparameters (Supplementary Table S3). The LightGBM model provided the best performance in the testing set: AUROC (0.912), AUPRC (0.915), accuracy (0.843), precision (0.843), recall (0.842), F1 score (0.843), sensitivity (0.842), and specificity (0.844). Similarly, clinical decision curve analysis (DCA) also demonstrated that the LightGBM model outperformed the other nine models (Fig. 3c).

**Table 2:** Predictive performances of the ten ML models in the testing set^*^

Model	Accuracy	Precision	Recall	F1	Sensitivity	Specificity	AUROC	AUPRC
Logistic Regression	0.802(0.780 ~ 0.824)	0.811(0.780 ~ 0.840)	0.788(0.756 ~ 0.821)	0.799(0.774 ~ 0.822)	0.788(0.756 ~ 0.821)	0.816(0.786 ~ 0.845)	0.870(0.852 ~ 0.889)	0.880(0.856 ~ 0.901)
Random Forest	0.833(0.813 ~ 0.854)	0.837(0.807 ~ 0.865)	0.827(0.798 ~ 0.857)	0.832(0.809 ~ 0.855)	0.827(0.798 ~ 0.857)	0.839(0.811 ~ 0.867)	0.906(0.890 ~ 0.923)	0.912(0.893 ~ 0.930)
KNN	0.797(0.775 ~ 0.819)	0.791(0.761 ~ 0.823)	0.806(0.777 ~ 0.836)	0.798(0.775 ~ 0.823)	0.806(0.777 ~ 0.836)	0.788(0.756 ~ 0.818)	0.848(0.827 ~ 0.869)	0.862(0.839 ~ 0.884)
Naive Bayes	0.767(0.745 ~ 0.791)	0.803(0.771 ~ 0.837)	0.707(0.671 ~ 0.742)	0.752(0.724 ~ 0.779)	0.707(0.671 ~ 0.742)	0.827(0.797 ~ 0.856)	0.854(0.832 ~ 0.873)	0.859(0.831 ~ 0.884)
SVM	0.809(0.788 ~ 0.830)	0.817(0.785 ~ 0.847)	0.795(0.765 ~ 0.826)	0.806(0.782 ~ 0.829)	0.795(0.765 ~ 0.826)	0.822(0.792 ~ 0.850)	0.868(0.847 ~ 0.888)	0.865(0.835 ~ 0.892)
XGBoost	0.833(0.813 ~ 0.854)	0.837(0.808 ~ 0.864)	0.827(0.798 ~ 0.857)	0.832(0.809 ~ 0.853)	0.827(0.798 ~ 0.857)	0.839(0.810 ~ 0.867)	0.912(0.896 ~ 0.927)	**0.915(0.897 ~ 0.932)**
AdaBoost	0.802(0.780 ~ 0.822)	0.801(0.769 ~ 0.830)	0.804(0.774 ~ 0.833)	0.802(0.777 ~ 0.825)	0.804(0.774 ~ 0.833)	0.800(0.768 ~ 0.829)	0.882(0.864 ~ 0.900)	0.871(0.841 ~ 0.899)
LightGBM	**0.843(0.823 ~ 0.863)**	**0.843(0.813 ~ 0.870)**	**0.842(0.813 ~ 0.870)**	**0.843(0.820 ~ 0.863)**	**0.842(0.813 ~ 0.870)**	**0.844(0.815 ~ 0.872)**	0.912(0.896 ~ 0.927)	**0.915(0.894 ~ 0.932)**
Decision Tree	0.729(0.706 ~ 0.753)	0.724(0.690 ~ 0.758)	0.741(0.705 ~ 0.773)	0.732(0.704 ~ 0.760)	0.741(0.705 ~ 0.773)	0.718(0.682 ~ 0.753)	0.729(0.706 ~ 0.754)	0.797(0.774 ~ 0.820)
Gradient Boosting	0.832(0.811 ~ 0.853)	0.830(0.799 ~ 0.857)	0.836(0.808 ~ 0.864)	0.833(0.810 ~ 0.854)	0.836(0.808 ~ 0.864)	0.829(0.798 ~ 0.858)	**0.913(0.897 ~ 0.928)**	0.908(0.885 ~ 0.929)

Bold numbers are the highest values

^*^Values in parentheses are 95% Cis

### External validation of the LightGBM model

**Fig. 4:**
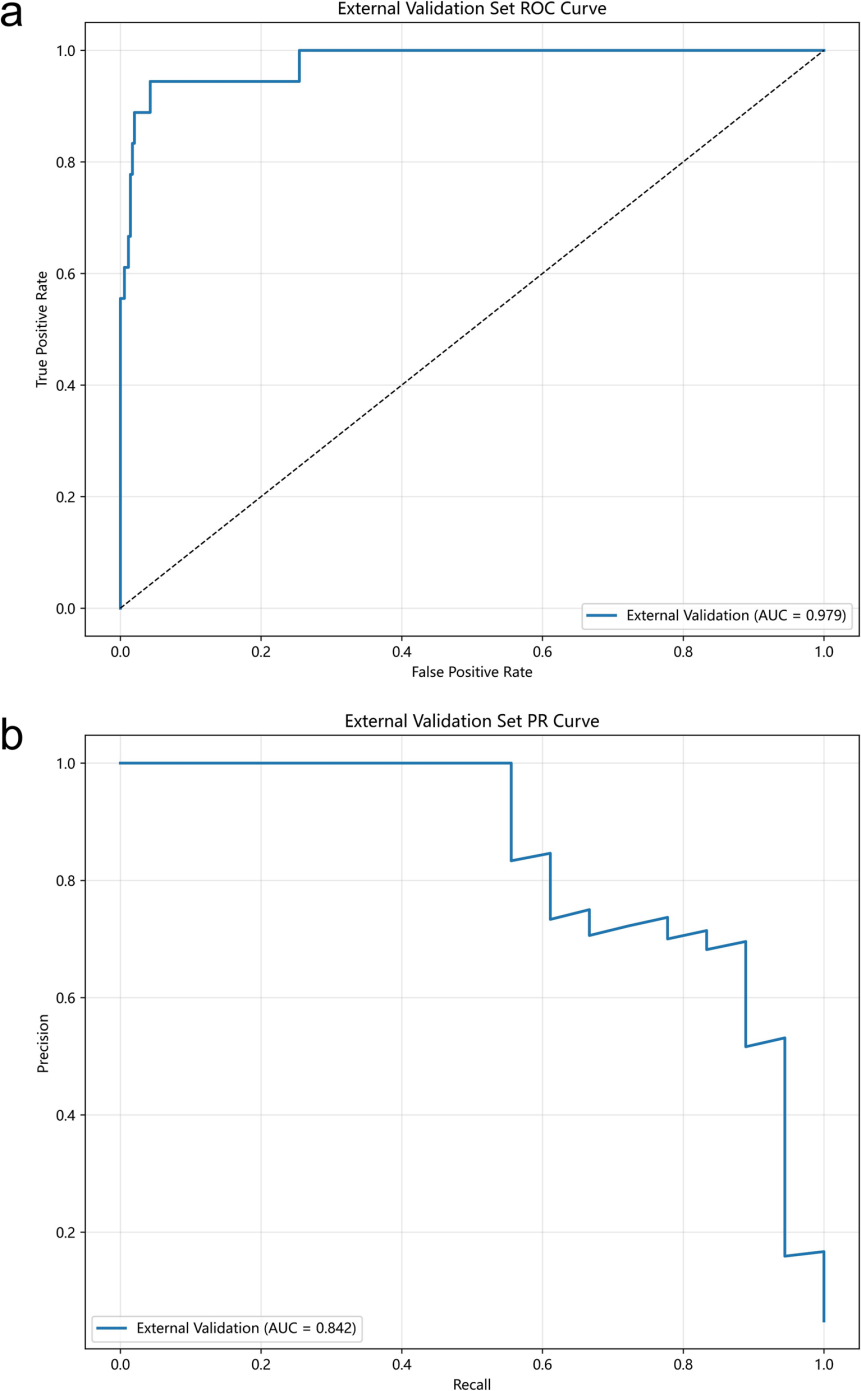
Performance of the LightGBM in prediction of severe infection in the external validation set based on AUROC **a** and AUPRC **b**

We then evaluated the generalizability of the LightGBM model by evaluating its performance in the external validation cohort. The results indicated excellent performance, with an AUROC of 0.979 and an AUPRC of 0.842 (Fig. 4a, b).

### Interpretation of the LightGBM model

Clinicians are usually reluctant to accept a prediction model that lacks a clinical interpretation. We therefore used the SHAP method to estimate the contribution of each of the ten variables to the predictions of the LightGBM model. This approach provides a global interpretation for different variables and a local interpretation for individual patients.

**Fig. 5:**
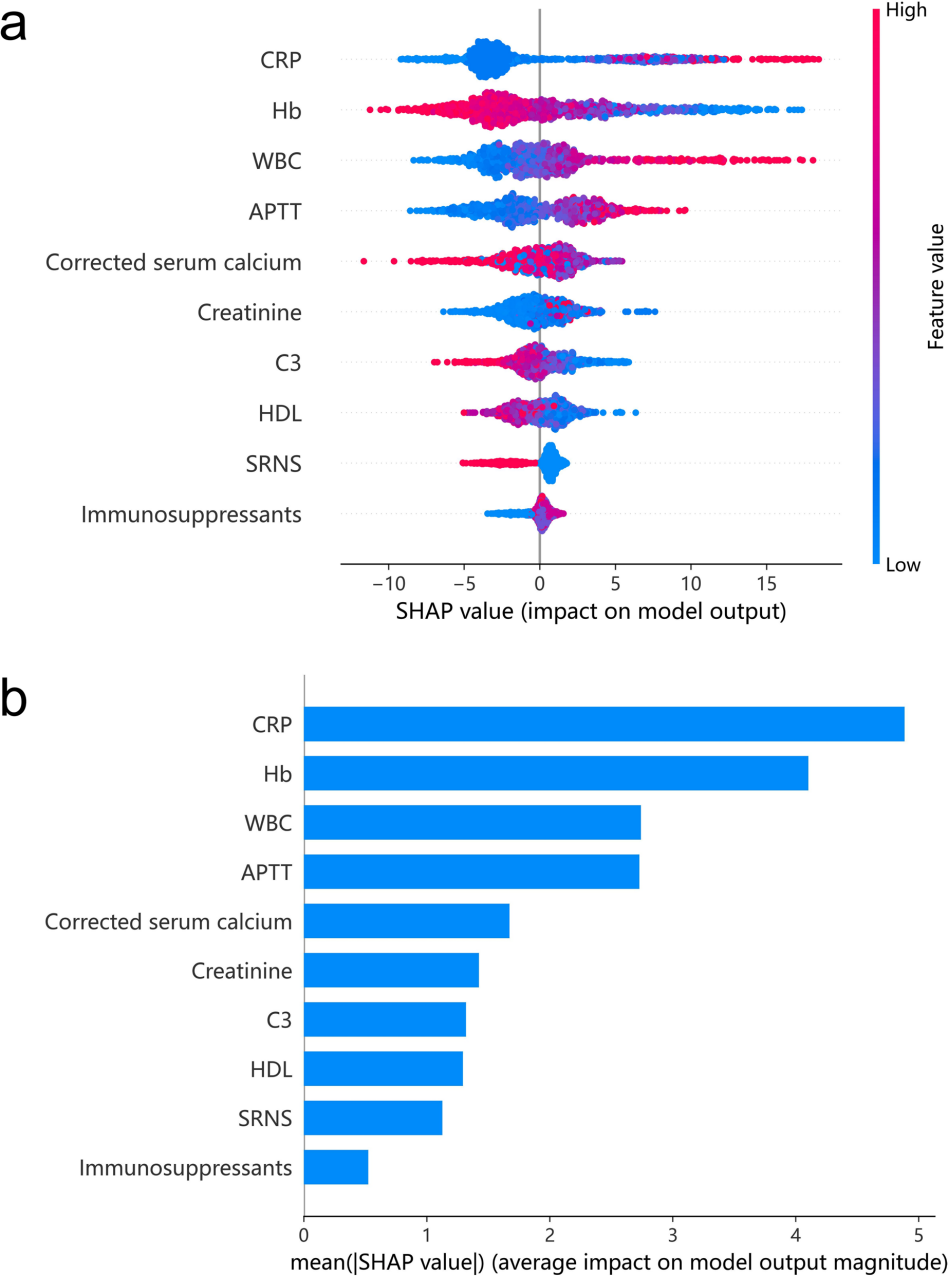
Global interpretation of the LightGBM based on the SHAP method, with presentation as a summary dot plot **a** and importance score **b**. Each point for each variable in (a) represents a single patient, with red representing a greater value and blue representing a lower value. A SHAP value above 0 indicates an increased risk of severe infection, a higher SHAP value indicates more risk, and a variable with a greater dispersion of points indicates a more significant impact on the model. CRP: C-reaction protein; WBC: white blood cell count; Hb: hemoglobin; APTT: activated partial thromboplastin time; HDL: high-density lipoprotein; C3: complement 3; SRNS: steroid-resistant nephrotic syndrome

At the global level, each point in the resulting summary dot plot represents the SHAP value of a single patient for each of the ten variables, with a red point representing a greater value and a blue point representing a lower value (Fig. 5a). A higher SHAP value represents greater risk. We also assessed the importance of the ten variables by plotting the mean SHAP values in descending order (Fig. 5b). Taken together, these results indicate that CRP and Hb had the most significant impact on model performance, because they had large ranges of SHAP values (Fig. 5a) and high mean SHAP values (Fig. 5b). We also provided SHAP dependence plot in Supplementary Figure S2.

**Fig. 6:**
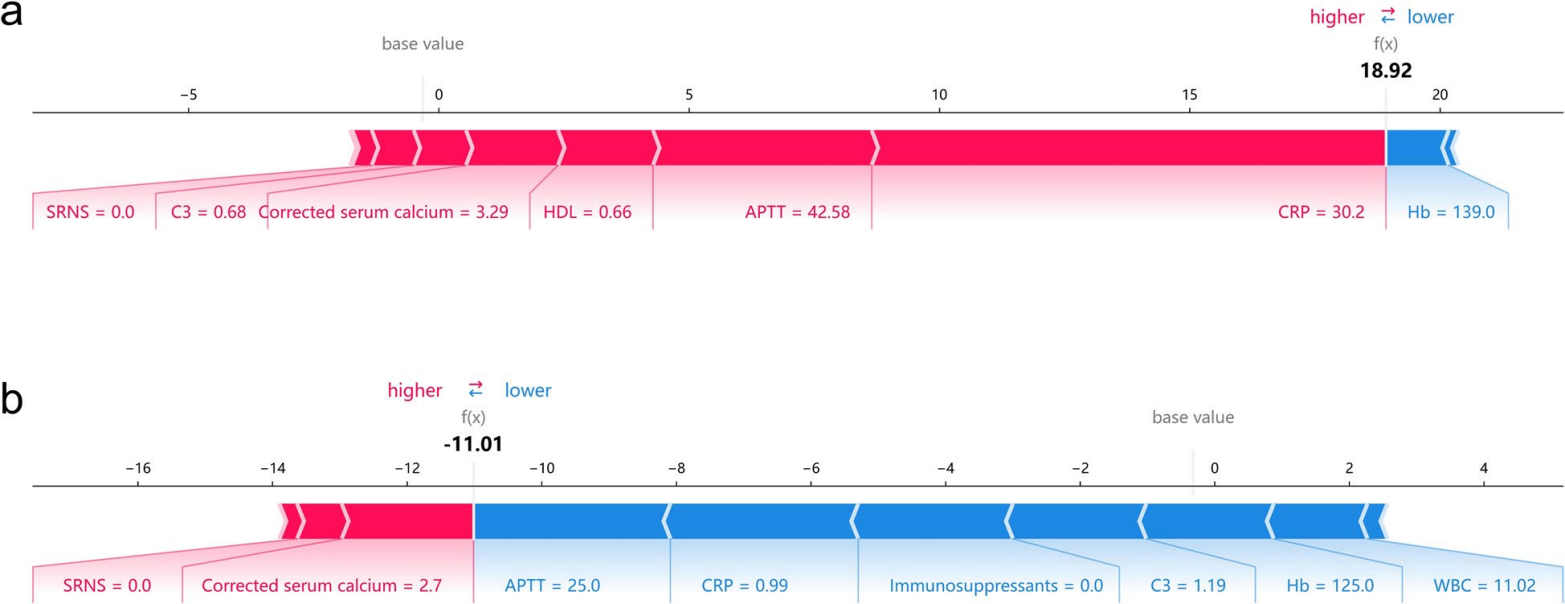
Force Plots showing the contribution of different variables to model output for prediction of severe infection in a representative patient with a severe infection **a** and in another representative patient with a non-severe infection **b**. Elevated CRP, decreased HDL, increased APTT, and increased WBC were the major features contributing to an increased risk of severe infection. A decreased value of APTT, high C3, normal Hb, the use of fewer immunosuppressants, low CRP, and low WBC were the major features contributing to a decreased risk of severe infection. CRP: C-reaction protein; WBC: white blood cell count; Hb: hemoglobin; APTT: activated partial thromboplastin time; HDL: high-density lipoprotein; C3: complement 3; Total Ca: total serum calcium

We presented these results as SHAP force plots for an individual INS patient who had a severe infection (Fig. 6a) and another INS patient who had a non-severe infection (Fig. 6b). These results emphasize that a high CRP, low HDL, high APTT, predicted a greater risk of severe infection, and that a low APTT, high C3, normal Hb, use of fewer immunosuppressants, a low CRP, and a low WBC predicted a lower risk of severe infection. Thus, the clinical interpretation of this ML model is that a patient with a poorer status based on ten clinical variables had a greater risk of severe infection.

**Fig. 7:**
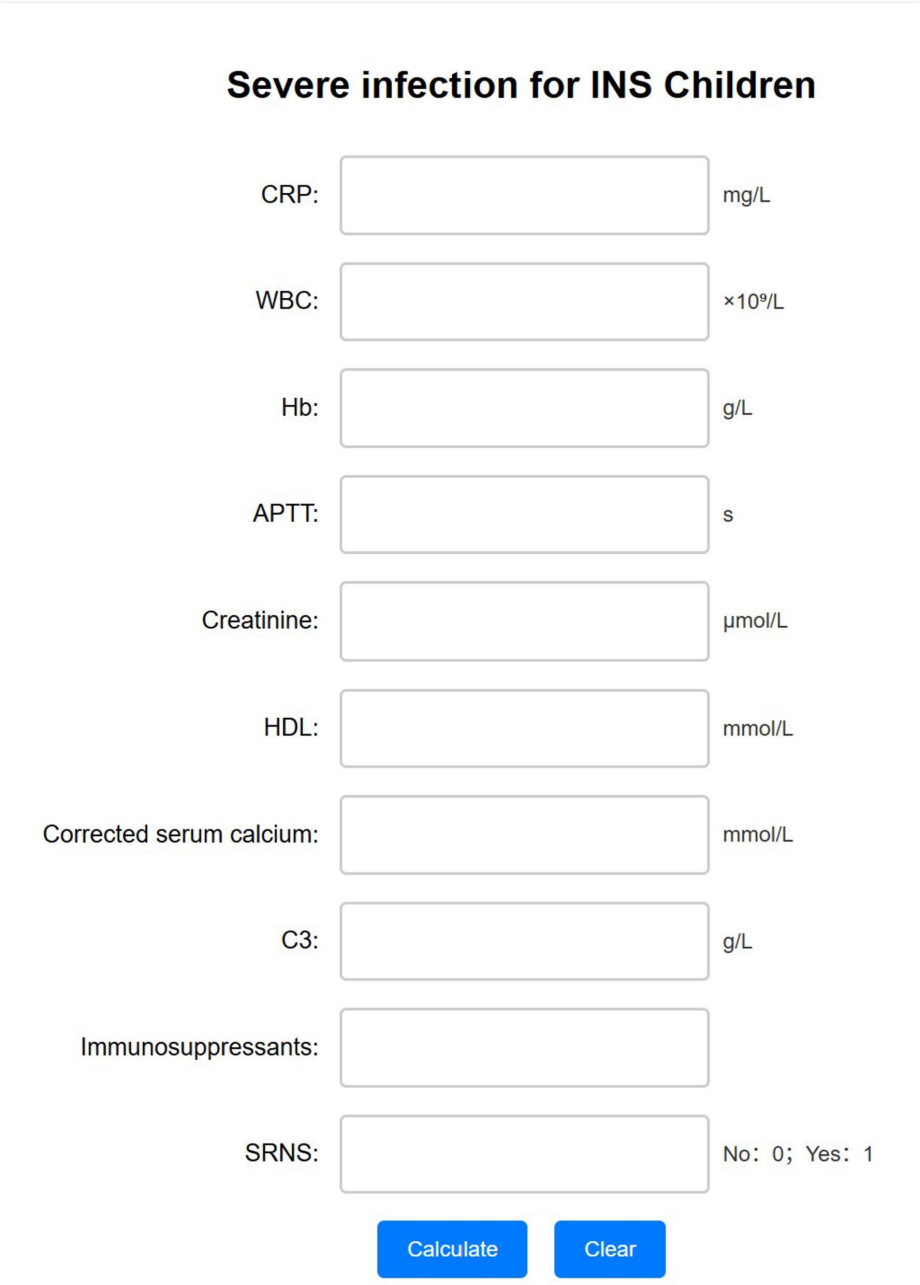
Optimized LightGBM model as a user-friendly web-based clinical decision support tool. A user enters the values of ten clinical variables of a child with INS, and the web tool automatically predicts the probability of severe infection. This online tool can be accessed at http://94.191.23.246:5004

The optimized LightGBM model was operationalized as a user-friendly web-based clinical decision support tool (Fig. 7). A user can enter the values of ten clinical variables of a child with INS, and the web tool automatically predicts the probability of severe infection. This model is deployed on the electronic medical system of the Chongqing Medical University Children’s Hospital and will be continuously updated to provide improved predictability and applicability as more patients are added to the research cohort. This online tool can be accessed at http://94.191.23.246:5004.

We also conducted a univariate analysis to explore whether these ten variables were associated with poor outcome (Death) in patients with severe infections. The results showed that a longer APTT, higher corrected serum calcium, lower HDL, and higher creatinine level were associated with poor outcome (Supplementary Table S4).

## Discussion

The recurrent and prolonged nature of INS, the high risk of infection due to treatment with immunosuppressants, the progression and different developmental stages of this disease, and the unique immune status of these children impose significant challenges for treatment. During the early stages of a stealth infection, symptoms may be atypical, thus complicating diagnosis. However, early diagnosis is critical because these infections can lead to disease recurrence and drug resistance [32]. Therefore, early identification of risk factors for severe infection in children with INS may facilitate personalized management strategies that lead to disease remission and improved quality-of-life.

The clinical significance of severe infection in children with INS motivated us to conduct a multicenter retrospective study to analyze the clinical characteristics of patients with INS complicated by severe infection. Given the great potential of ML algorithms for clinical science, we examined the effect of ten different ML models to identify factors that were best able to predict severe infection in INS patients. The LightGBM model had excellent predictive performance in the testing set (AUROC: 0.912, AUPRC: 0.915) and the external validation set (AUROC: 0.979, AUPRC: 0.842). Moreover, the final model only included ten readily available variables that are routinely measured in clinical settings, demonstrating the potential of this model in hospitals.

Although previous studies have analyzed risk factors for infection in children with INS, they did not use rigorous and systematic methods for model construction. Improvements in the early diagnosis of sepsis in children are due to the use of overall assessments and composite scoring models. Our model followed this pattern, in that we considered clinical variables related to coagulation, circulation, immunity, and lipid metabolism, rather than merely blood indicators of inflammation. Thus, our approach is in alignment with the Phoenix Sepsis Score (PSS) and the Phoenix-8 Sepsis Score [26].

When infection occurs, a decrease in the Hb level is common, and true or pseudo-anemia may also occur due to reduced erythropoiesis, shortened lifespan and increased destruction of RBCs, and hepcidin-mediated dysfunction of iron metabolism due to the systemic inflammatory response [33, 34]. When nephrotic syndrome recurs due to re-damage of the glomerular filtration barrier, patients will experience massive proteinuria and hypoalbuminemia, which leads to fluid retention and aggravated edema. This increase in fluid load causes weight gain, exacerbates edema, and also leads to blood dilution, further affecting the clinical manifestations and laboratory indicators of anemia [35]. Although there have been reports of decreased Hb levels in patients with sepsis and severe COVID-19 [36, 37], no studies in China or elsewhere have evaluated the use of the Hb decline as an indicator of infection severity. Our results showed that the Hb level was significantly lower in INS patients with severe infection. Further studies with larger sample sizes and in different populations are needed to confirm these findings.

The APTT is an effective indicator of coagulation function, in that it reflects the function of the intrinsic coagulation system, and is therefore widely employed for screening and assessments of deficiencies in coagulation. Previous research demonstrated that the APTT was significantly longer in patients with the sepsis [38], and sepsis is considered a primary cause of coagulation dysfunction. When an imbalance between procoagulant and anticoagulant systems leads to dysfunction of the secondary fibrinolytic system, diffuse intravascular coagulation (DIC) may lead to adverse clinical outcomes [39]. In line with these findings, it is crucial to emphasize the continuous monitoring of coagulation indices to enable early detection of severe infections based on APTT.

Previous studies have identified severe edema, ascites blood albumin below 15 g/L, blood cholesterol higher than 400 mg/dL, and the use of a combination of glucocorticoids and immunosuppressants as factors associated with infection in patients with INS [15, 40, 41]. We also identified factors associated with severe infection in children with INS, and these included increased levels of WBC, CRP, and creatinine, and use of more types of immunosuppressants. CRP, an acute-phase protein, begins to increase at 4 to 6 h after the onset of bacterial infection, and peaks at about 24 to 48 h. Although it only has moderate specificity for early identification of sepsis, previous studies showed that markedly elevated CRP level was associated with an increased risk of septic shock in children. Persistently elevated CRP often indicates inadequate infection control or the presence of complications [42]. Dynamic monitoring of CRP [43] and measurement of more specific biomarkers, such as procalcitonin and interleukin-6, can facilitate early detection of disease [44]. Future studies with larger sample sizes are needed to examine the use of these parameters as an early warning of infection. Although the median CRP levels were similar between the groups with severe and non-severe infections in our model—there were significant differences in their 95% CIs. This suggests that some patients in the severe infection group had inadequate infection control or other complications. Despite its moderate specificity for early identification of sepsis, we still used CRP as an important variable in our model for prediction of severe infection.

Madsen et al. [45] found that HDL had anti-inflammatory functions, and that a lower HDL level is associated with a greater risk of infection. This is consistent with the results of the present study, although other studies reported contrary findings [46]. Nevertheless, our results demonstrated that a high HDL level was negatively associated with severe infection in children with INS.

A 2022 study by di Filippo et al. showed that the incidence of hypocalcemia was higher in patients with COVID-19 and that hypocalcemia was associated with disease severity and patient mortality [47]. A 2019 study of sepsis patients showed that the blood calcium level was lower and the APACHE-II score was higher in those who died than in the survivors. This 2019 study also reported the AUROC for blood calcium in predicting patient prognosis was 0.70, indicating a decreased blood calcium level can be considered a warning of severe infection [48]. The body maintains the serum calcium concentration within an extremely narrow range, and blood calcium exists as ionized and bound forms; only the ionized form is biologically active and has physiological effects. However, because ionized calcium can be accessed via arterial or venous sampling or estimated from total calcium and albumin levels, we employed corrected calcium in our cohort to evaluate its predictive value in severe infections.

The complement system releases C3a and C5a, which facilitate the migration of white blood cells to sites of inflammation as part of the inflammatory response. Simultaneously, complement activation mediates the formation of membrane attack complexes, thus contributing to cellular damage. Previous studies [49] suggested a correlation between the complement C3 level and disease progression and prognosis in pediatric sepsis, and this is supported by our preliminary findings. In particular, we observed lower average C3 level in children with severe infection than in those with non-severe infection. However, the nature of this association should be considered tentative. Further validation in large and well-designed studies is needed to clarify the association of C3 with infection severity and its potential as a prognostic biomarker.

Previous studies reported that renal insufficiency was a risk factor for infection in patients undergoing immunosuppressive therapy [50]. Due to the decreased glomerular filtration rate (GFR) in patients with renal disease, immunosuppressants will accumulate in the circulation. Research has indicated that patients with severe renal insufficiency have compromised cellular immune function, with a decreased CD4/CD8 ratio, an elevated Th1/Th2 ratio, and exhaustion of CD4 + and CD8 + T cells [51]. These patients also experience decreased phagocytic functions of granulocytes and monocytes/macrophages, incomplete antigen-presenting capacity of antigen-presenting cells, a decreased number of B lymphocytes, and a decreased ability to produce antibodies, all of which can increase the risk of infection [52, 53]. Patients with renal insufficiency have a greater risk of infection than those with normal renal function [54]. This is in accordance with our findings that risk factors for severe infection were elevated serum creatinine and use of more immunosuppressants.

This study described the development of a ML model for predicting severe infection in patients with INS in an aim to facilitate earlier diagnosis and treatment of severe infections in these patients. We conducted the first systematic analysis of immunosuppressant effects in INS patients and evaluated their value in predicting severe infection. Our finding that increased use of immunosuppressants was associated with higher risk of severe infection, thus underscoring the need for careful selection of treatment. It should be noted that different immunosuppressants act on distinct immune pathways, which may differentially influence infection risk. However, due to the retrospective design and limited data, our study could not fully account for these variations. Future studies should incorporate improved designs to address this issue.

In the effort to achieve remission from proteinuria, nephrologists should also consider individualized pharmacogenomic profiles. The growing availability of next-generation sequencing enables more precise assessment of drug metabolism and supports personalized and safer selection of immunosuppressants.

Our research has several key strengths. Firstly, we performed a multicenter study, with data from four pediatric centers that specialized in the diagnosis and treatment of kidney diseases in China. Secondly, we constructed a ML model based on a large sample size from a single center, and all clinical variables in this model were relatively common and easy to obtain. This means that nephrologists can easily use this model for the early identification of INS patients with severe infection. This will provide better management of these patients with limited resources, and decrease hospitalization time and medical expenses. Thirdly, we identified some novel key factors for predicting severe infection in INS patients: APTT, Hb and HDL. Further study of these factors may provide new clinical perspectives and guidelines regarding the treatment of INS patients.

However, this study also has several limitations. Firstly, all patients were from China. It is likely that the racial and genetic background can affect disease susceptibility and phenotype, and the response to different INS treatments. The sample size was mainly collected from the electronic medical record systems of hospitals in three provinces of China (Chongqing, Sichuan, and Jiangsu), and the sample size of some centers was quite small. Secondly, due to missing data in many patients, we were unable to incorporate certain important clinical variables. Thirdly, due to the limitations in sample collection, we were unable to obtain trough levels of immunosuppressant, and this could be useful in predicting severe infection. Fourthly, the very high AUROC values may be partially attributable to sample imbalance and the limited number of cases. Finally, the clinical applicability of the model requires external prospective validation with a larger and more diverse cohort.

## Conclusions

We developed a simple and interpretable ML model using routine clinical data from EMR systems to predict severe infection in children with INS. The model demonstrated excellent predictive performance during internal testing at CMUCH and also had promising results in external validation using data from three other hospitals. To our knowledge, this is the first multi-center study to apply ML for prediction of severe infection in a pediatric population and to identify key predictive factors.

However, we acknowledge that the retrospective, single-center design and the limited size of the external validation cohort limit the generalizability of our findings. Further validation in larger, multi-ethnic populations is necessary before clinical application can be widely recommended.

Despite these limitations, the model requires only ten commonly measured clinical features, and this facilitates it potential acceptance by nephrologists. This work highlights that AI and ML models can serve as valuable integrative tools—not replacements—for clinicians, particularly in complex conditions such as INS where nuanced diagnostic and timely therapeutic decisions are required. If validated prospectively, this approach could offer an effective, convenient, and cost-effective strategy for preventing severe infections in children with INS.

## Supplementary Information

Supplementary Material 1: Supplementary Figure S1. The percentages of missing data of variables. Supplementary Figure S2. SHAP dependence plot.

Supplementary Material 2: Supplementary Table S1. General variables and definitions of severe infection. Supplementary Table S2. Baseline characteristics of patients in the training, test and validation cohorts. Supplementary Table S3. Hyper-parameter settings of the proposed model. Supplementary Table S4. Univariate analysis of adverse outcomes in critically infected patients.

## Data Availability

The data analyzed and the codes used during the current study are available from the corresponding author on reasonable request.

## Abbreviations

INS: Idiopathic nephrotic syndrome
IPNA: International pediatric nephrology association
EMR: Electronic medical record; AI: artificial intelligence
ML: Machine learning
WBC: White blood cell
LYM%: Percentage of lymphocytes
NEU%: Percentage of neutrophils
AMC: Absolute monocyte count
Hb: Hemoglobin
PLT: Platelet count
CRP: C-reactive protein
TP: Total protein
ALB: Albumin
GLB: Globulin\
ALT: Alanine aminotransferase
GGT: Glutamyl transpeptidase
uRBC: Urine red blood cell count
TGs: Triglycerides
LDL: Low-density lipoprotein cholesterol
HDL: High-density lipoprotein cholesterol
LDH: Lactate dehydrogenase
Mg: Magnesium
Ca: Calcium
APTT: Activated partial thromboplastin time
TT: Thrombin time
IgG: Immunoglobulin G
IgE: Immunoglobulin E
IgA: Immunoglobulin A
IgM: Immunoglobulin M
C3: Complement 3
C4: Complement 4
SRNS: Steroid-resistant nephrotic syndrome
LASSO: Least absolute shrinkage and selection operator
UPCR: Urinary protein-creatinine ratio
ARDS: Acute respiratory distress syndrome
MODS: Multiple organ dysfunction syndrome
LR: Logistic Regression
RF: Random Forest
KNN: K-Nearest Neighbors
NB: Naïve Bayes
SVM: Support Vector Machine
XGBoost: Extreme gradient boosting; adaboost,adaptive boosting
LightGBM: Light gradient boosting machine
DT: Decision Tree
GBM: Gradient boosting machine
SMOTE: Synthetic minority over-sampling technique
AUROC: Area under the receiver operating characteristic curve
AUPRC: Area under the precision-recall curve
SHAP: SHapley Additive exPlanations

## References

[1] Veltkamp F, Rensma LR, Bouts AHM. Incidence and relapse of idiopathic nephrotic syndrome: meta-analysis. Pediatrics. 2021. 10.1542/peds.2020-029249. 34193618 10.1542/peds.2020-029249

[2] Vivarelli M, Gibson K, Sinha A, Boyer O. Childhood nephrotic syndrome. Lancet. 2023;402:809–24. 37659779 10.1016/S0140-6736(23)01051-6

[3] Trautmann A, Boyer O, Hodson E, Bagga A, Gipson DS, Samuel S, et al. IPNA clinical practice recommendations for the diagnosis and management of children with steroid-sensitive nephrotic syndrome. Pediatr Nephrol. 2023;38:877–919. 36269406 10.1007/s00467-022-05739-3 PMC9589698

[4] Zhang H, Qiu S, Zhong C, Shi L, Li J, Zhang T, et al. Risk factors for poor prognosis of severe infection in children with idiopathic nephrotic syndrome: a double-center, retrospective study. Front Pediatr. 2021;9:656215. 34336733 10.3389/fped.2021.656215 PMC8316585

[5] Latta K, von Schnakenburg C, Ehrich JH. A meta-analysis of cytotoxic treatment for frequently relapsing nephrotic syndrome in children. Pediatr Nephrol. 2001;16:271–82. 11322378 10.1007/s004670000523

[6] Noone DG, Iijima K, Parekh R. Idiopathic nephrotic syndrome in children. Lancet. 2018;392:61–74. 29910038 10.1016/S0140-6736(18)30536-1

[7] Han JW, Lee KY, Hwang JY, Koh DK, Lee JS. Antibody status in children with steroid-sensitive nephrotic syndrome. Yonsei Med J. 2010;51:239–43. 20191016 10.3349/ymj.2010.51.2.239 PMC2824870

[8] Cain DW, Cidlowski JA. Immune regulation by glucocorticoids. Nat Rev Immunol. 2017;17:233–47. 28192415 10.1038/nri.2017.1 PMC9761406

[9] Eddy AA, Symons JM. Nephrotic syndrome in childhood. Lancet. 2003;362:629–39. 12944064 10.1016/S0140-6736(03)14184-0

[10] Wei CC, Yu IW, Lin HW, Tsai AC. Occurrence of infection among children with nephrotic syndrome during hospitalizations. Nephrology. 2012;17:681–8. 22882426 10.1111/j.1440-1797.2012.01650.x

[11] Liponski I, Cochat P, Gagnadoux MF, Parchoux B, Niaudet P, David L, et al. Bacterial complications of nephrotic syndrome in children. Presse Med. 1995;24:19–22. 7899329

[12] Alwadhi RK, Mathew JL, Rath B. Clinical profile of children with nephrotic syndrome not on glucorticoid therapy, but presenting with infection. J Paediatr Child Health. 2004;40:28–32. 14718000 10.1111/j.1440-1754.2004.00285.x

[13] Hassan I, Tiewsoh JBA, Ray P, Dawman L, Rathore V, Suri D, et al. Changing spectrum of infections in childhood nephrotic syndrome. Indian J Pediatr. 2019;86:1065. 31209762 10.1007/s12098-019-03007-1

[14] Glenn DA, Henderson CD, O’Shaughnessy M, Hu Y, Bomback A, Gibson K, et al. Infection-related acute care events among patients with glomerular disease. Clin J Am Soc Nephrol. 2020;15:1749–61. 33082200 10.2215/CJN.05900420 PMC7769021

[15] Kumar M, Ghunawat J, Saikia D, Manchanda V. Incidence and risk factors for major infections in hospitalized children with nephrotic syndrome. Braz J Nephrol. 2019;41:526–33. 10.1590/2175-8239-JBN-2019-0001 PMC6979567 31528983

[16] Sidey-Gibbons JAM, Sidey-Gibbons CJ. Machine learning in medicine: a practical introduction. BMC Med Res Methodol. 2019;19:64. 30890124 10.1186/s12874-019-0681-4 PMC6425557

[17] Collins GS, Moons KGM, Dhiman P, Riley RD, Beam AL, Van Calster B, et al. TRIPOD+AI statement: updated guidance for reporting clinical prediction models that use regression or machine learning methods. BMJ. 2024;385:e078378. 38626948 10.1136/bmj-2023-078378 PMC11019967

[18] Fleuren LM, Klausch TLT, Zwager CL, Schoonmade LJ, Guo T, Roggeveen LF, et al. Machine learning for the prediction of sepsis: a systematic review and meta-analysis of diagnostic test accuracy. Intensive Care Med. 2020;46:383–400. 31965266 10.1007/s00134-019-05872-y PMC7067741

[19] Giannini HM, Ginestra JC, Chivers C, Draugelis M, Hanish A, Schweickert WD, et al. A machine learning algorithm to predict severe sepsis and septic shock: development, implementation, and impact on clinical practice. Crit Care Med. 2019;47:1485–92. 31389839 10.1097/CCM.0000000000003891 PMC8635476

[20] Yang T, Zhang L, Sun S, Yao X, Wang L, Ge Y. Identifying severe community-acquired pneumonia using radiomics and clinical data: a machine learning approach. Sci Rep. 2024;14:21884. 39300101 10.1038/s41598-024-72310-5 PMC11413163

[21] Abujaber AA, Yaseen S, Nashwan AJ, Akhtar N, Imam Y. Prediction of stroke-associated hospital-acquired pneumonia: machine learning approach. J Stroke Cerebrovasc Dis. 2025;34:108200. 39674434 10.1016/j.jstrokecerebrovasdis.2024.108200

[22] Rattan P, Penrice DD, Simonetto DA. Artificial intelligence and machine learning: what you always wanted to know but were afraid to ask. Gastro Hep Advances. 2022;1:70–8. 39129929 10.1016/j.gastha.2021.11.001 PMC11307451

[23] Hou F, Zhu Y, Zhao H, Cai H, Wang Y, Peng X, et al. Development and validation of an interpretable machine learning model for predicting the risk of distant metastasis in papillary thyroid cancer: a multicenter study. E Clin Med. 2024;77:102913. 10.1016/j.eclinm.2024.102913 PMC11567106 39552714

[24] Efthimiou O, Seo M, Chalkou K, Debray T, Egger M, Salanti G. Developing clinical prediction models: a step-by-step guide. BMJ. 2024;386:e078276. 39227063 10.1136/bmj-2023-078276 PMC11369751

[25] Liu X, Xie Z, Zhang Y, Huang J, Kuang L, Li X, et al. Machine learning for predicting in-hospital mortality in elderly patients with heart failure combined with hypertension: a multicenter retrospective study. Cardiovasc Diabetol. 2024;23:407. 39548495 10.1186/s12933-024-02503-9 PMC11568583

[26] Schlapbach LJ, Watson RS, Sorce LR, Argent AC, Menon K, Hall MW, et al. International consensus criteria for pediatric sepsis and septic shock. JAMA. 2024;331:665–74. 38245889 10.1001/jama.2024.0179 PMC10900966

[27] Li J, Zhang Q, Su B. Clinical characteristics and risk factors of severe infections in hospitalized adult patients with primary nephrotic syndrome. J Int Med Res. 2017;45:2139–45. 28661269 10.1177/0300060517715339 PMC5805218

[28] Bunkhumpornpat C, Boonchieng E, Chouvatut V, Lipsky D. Flex-smote: Synthetic over-sampling technique that flexibly adjusts to different minority class distributions. Patterns. 2024;5:101073. 39568474 10.1016/j.patter.2024.101073 PMC11573909

[29] Hu J, Xu J, Li M, Jiang Z, Mao J, Feng L, et al. Identification and validation of an explainable prediction model of acute kidney injury with prognostic implications in critically ill children: a prospective multicenter cohort study. EClinicalMedicine. 2024;68:102409. 38273888 10.1016/j.eclinm.2023.102409 PMC10809096

[30] Lundberg SM, Erion G, Chen H, DeGrave A, Prutkin JM, Nair B, et al. From local explanations to global understanding with explainable AI for trees. Nat Mach Intell. 2020;2:56–67. 32607472 10.1038/s42256-019-0138-9 PMC7326367

[31] Nohara Y, Matsumoto K, Soejima H, Nakashima N. Explanation of machine learning models using shapley additive explanation and application for real data in hospital. Comput Methods Programs Biomed. 2022;214:106584. 34942412 10.1016/j.cmpb.2021.106584

[32] Lin CH, Hung PH, Liu WS, Hu HY, Chung CJ, Chen TH. Infections and risk of end-stage renal disease in patients with nephrotic syndrome: a nationwide population-based case-control study. Ann Transl Med. 2020;8:228. 32309375 10.21037/atm.2020.01.02 PMC7154467

[33] Rogiers P, Zhang H, Leeman M, Nagler J, Neels H, Mélot C, et al. Erythropoietin response is blunted in critically ill patients. Intensive Care Med. 1997;23:159–62. 9069000 10.1007/s001340050310

[34] Ganz T. Anemia of inflammation. N Engl J Med. 2019;381:1148–57. 31532961 10.1056/NEJMra1804281

[35] Ellis D. Pathophysiology, evaluation, and management of edema in childhood nephrotic syndrome. Front Pediatr. 2015;3:111. 26793696 10.3389/fped.2015.00111 PMC4707228

[36] Vincent JL, Baron JF, Reinhart K, Gattinoni L, Thijs L, Webb A, et al. Anemia and blood transfusion in critically ill patients. JAMA. 2002;288:1499–507. 12243637 10.1001/jama.288.12.1499

[37] Chen N, Zhou M, Dong X, Qu J, Gong F, Han Y, et al. Epidemiological and clinical characteristics of 99 cases of 2019 novel coronavirus pneumonia in Wuhan, China: a descriptive study. Lancet. 2020;395:507–13. 32007143 10.1016/S0140-6736(20)30211-7 PMC7135076

[38] Mohapatra P, Kumar A, Singh RK, Gupta R, Hussain M, Singh S, et al. The effect of sepsis and septic shock on the viscoelastic properties of clot quality and mass using thromboelastometry: a prospective observational study. Indian J Crit Care Med. 2023;27:625–34. 37719352 10.5005/jp-journals-10071-24539 PMC10504658

[39] Rinaldi I, Sudaryo MK, Prihartono NA. Disseminated intravascular coagulation in sepsis and associated factors. J Clin Med. 2022. 10.3390/jcm11216480. 36362708 10.3390/jcm11216480 PMC9658286

[40] Ajayan P, Krishnamurthy S, Biswal N, Mandal J. Clinical spectrum and predictive risk factors of major infections in hospitalized children with nephrotic syndrome. Indian Pediatr. 2013;50:779–81. 23502669 10.1007/s13312-013-0214-x

[41] Alfakeekh K, Azar M, Sowailmi BA, Alsulaiman S, Makdob SA, Omair A, et al. Immunosuppressive burden and risk factors of infection in primary childhood nephrotic syndrome. J Infect Public Health. 2019;12:90–4. 30279098 10.1016/j.jiph.2018.09.006

[42] Lamping F, Jack T, Rübsamen N, Sasse M, Beerbaum P, Mikolajczyk RT, et al. Development and validation of a diagnostic model for early differentiation of sepsis and non-infectious SIRS in critically ill children - a data-driven approach using machine-learning algorithms. BMC Pediatr. 2018;18:112. 29544449 10.1186/s12887-018-1082-2 PMC5853156

[43] Lanziotti VS, Póvoa P, Prata-Barbosa A, Pulcheri LB, Rabello L, Lapa ESJR, et al. Patterns of C-reactive protein ratio response to antibiotics in pediatric sepsis: a prospective cohort study. J Crit Care. 2018;44:217–22. 29161668 10.1016/j.jcrc.2017.11.018

[44] Wu Y, Wang G, Huang Z, Yang B, Yang T, Liu J, et al. Diagnostic and therapeutic value of biomarkers in urosepsis. Ther Adv Urol. 2023;15:17562872231151852. 36744043 10.1177/17562872231151852 PMC9893402

[45] Madsen CM, Varbo A, Tybjærg-Hansen A, Frikke-Schmidt R, Nordestgaard BG. U-shaped relationship of HDL and risk of infectious disease: two prospective population-based cohort studies. Eur Heart J. 2018;39:1181–90. 29228167 10.1093/eurheartj/ehx665

[46] Speer T, Zewinger S. High-density lipoprotein (HDL) and infections: a versatile culprit. Eur Heart J. 2018;39:1191–3. 29240892 10.1093/eurheartj/ehx734

[47] di Filippo L, Doga M, Frara S, Giustina A. Hypocalcemia in COVID-19: prevalence, clinical significance and therapeutic implications. Rev Endocr Metab Disord. 2022;23:299–308. 33846867 10.1007/s11154-021-09655-z PMC8041474

[48] Fei M, Li P, Tao X, Pan A. Influence of hypocalcemia on the prognosis of septic patients. Wang J Zhonghua Wei Zhong Bing Ji Jiu Yi Xue. 2019;31:418–21. 31109413 10.3760/cma.j.issn.2095-4352.2019.04.009

[49] Mannes M, Schmidt CQ, Nilsson B, Ekdahl KN, Huber-Lang M. Complement as driver of systemic inflammation and organ failure in trauma, burn, and sepsis. Semin Immunopathol. 2021;43:773–88. 34191093 10.1007/s00281-021-00872-x PMC8243057

[50] Ye WL, Tang N, Wen YB, Li H, Li MX, Du B, et al. Underlying renal insufficiency: the pivotal risk factor for *Pneumocystis jirovecii* pneumonia in immunosuppressed patients with non-transplant glomerular disease. Int Urol Nephrol. 2016;48:1863–71. 27351666 10.1007/s11255-016-1324-x

[51] Vaziri ND, Pahl MV, Crum A, Norris K. Effect of uremia on structure and function of immune system. J Ren Nutr. 2012;22:149–56. 22200433 10.1053/j.jrn.2011.10.020 PMC3246616

[52] Massry S, Smogorzewski M. Dysfunction of polymorphonuclear leukocytes in uremia: role of parathyroid hormone. Kidney Int Suppl. 2001;78:S195–6. 11169010 10.1046/j.1523-1755.2001.59780195.x

[53] Smogorzewski M, Massry SG. Defects in B-cell function and metabolism in uremia: role of parathyroid hormone. Kidney Int Suppl. 2001;78:S186–9. 11169008 10.1046/j.1523-1755.2001.59780186.x

[54] Han F, Gao J, Gai J. Detection of myocardial enzymes, cardiac troponin T and hepatic and renal function in the diagnosis and treatment of severe pneumonia in children. Pak J Med Sci. 2018;34:1257–61. 30344587 10.12669/pjms.345.15397 PMC6191768

