# Supplementary figures and images for: Machine learning model for predicting severe infection in children with idiopathic nephrotic syndrome: multicenter retrospective study
Source: Ital J Pediatr. 2025 Nov 25;51:308. doi: 10.1186/s13052-025-02149-7 (PMC12648841; doi:10.1186/s13052-025-02149-7)

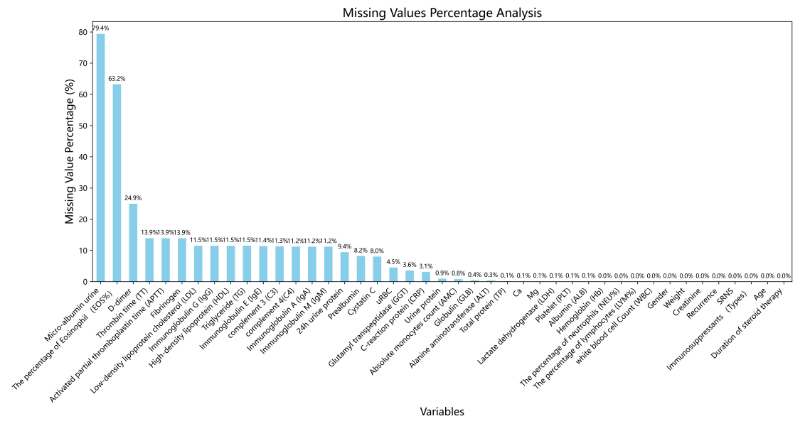

Supplement: Supplementary file 1 — Supplementary Material 1: Supplementary Figure S1. The percentages of missing data of variables. Supplementary Figure S2. SHAP dependence plot. [file 13052_2025_2149_MOESM1_ESM.zip › Supplementary Figure S1.tif]
