# Supplementary material for: Machine learning model for predicting severe infection in children with idiopathic nephrotic syndrome: multicenter retrospective study
Source: Ital J Pediatr. 2025 Nov 25;51:308. doi: 10.1186/s13052-025-02149-7 (PMC12648841; doi:10.1186/s13052-025-02149-7)
Supplement: Supplementary file 2 — Supplementary Material 2: Supplementary Table S1. General variables and definitions of severe infection. Supplementary Table S2. Baseline characteristics of patients in the training, test and validation cohorts. Supplementary Table S3. Hyper-parameter settings of the proposed model. Supplementary Table S4. Univariate analysis of adverse outcomes in critically infected patients. [file 13052_2025_2149_MOESM2_ESM.zip › Supplementary Table S1.docx]

**Supplementary Table S1 General variables and definitions of severe infection.**

| **General signs** |  |
| --- | --- |
| Fever (>38.5°C) | |
| Hypothermia (<35°C) | |
| Heart rate more than two SD above the normal value for age | |
| Tachypnea (Respiratory rate higher than WHO classification for age) | |
| Altered mental status | |
| **Inflammatory variables** |  |
| Leukocytosis (WBC count > 12,000 μL−1 or higher than the normal value for age) | |
| Leukopenia (WBC count <4,000 μL−1 or below the normal value for age) | |
| Plasma C-reactive protein more than two SD above the normal value | |
| Plasma procalcitonin more than two SD above the normal value | |
| **Hemodynamic variables** |  |
| Arterial hypotension (SBP less than two SD below normal value for age) | |
| **Organ dysfunction variables** |  |
| Arterial hypoxemia (PaO2/FiO2 <300) | |
| Acute oliguria (urine output <0.5 mL kg−1 h−1 for at least 2 h despite adequate fluid resuscitation) | |
| Coagulation abnormalities | |
| Ileus (absent bowel sounds) | |
| **Tissue perfusion variables** | |
| Hyperlactatemia (Lactate above upper limits laboratory normal) | |
| Decreased capillary refill or mottling | |
| **Phoenix sepsis criteria** | |
| Suspected infection and Phoenix Sepsis Score ≥2 points | |
