# Supplementary material for: Machine learning model for predicting severe infection in children with idiopathic nephrotic syndrome: multicenter retrospective study
Source: Ital J Pediatr. 2025 Nov 25;51:308. doi: 10.1186/s13052-025-02149-7 (PMC12648841; doi:10.1186/s13052-025-02149-7)
Supplement: Supplementary file 2 — Supplementary Material 2: Supplementary Table S1. General variables and definitions of severe infection. Supplementary Table S2. Baseline characteristics of patients in the training, test and validation cohorts. Supplementary Table S3. Hyper-parameter settings of the proposed model. Supplementary Table S4. Univariate analysis of adverse outcomes in critically infected patients. [file 13052_2025_2149_MOESM2_ESM.zip › Supplementary Table S3.docx]

| **Model** | **Optimal hyperparameters** |
| --- | --- |
| Logistic Regression | {'logisticregression__C': 0.1, 'logisticregression__penalty': 'l2'} |
| Random Forest | {'max_depth': None, 'min_samples_split': 2, 'n_estimators': 200} |
| KNN | {'kneighborsclassifier__n_neighbors': 3, 'kneighborsclassifier__p': 1} |
| Naive Bayes | {var_smoothing=1e-09} |
| SVM | {'svc__C': 10, 'svc__gamma': 'scale', 'svc__kernel': 'rbf'} |
| XGBoost | {'learning_rate': 0.2, 'max_depth': 7, 'n_estimators': 100} |
| AdaBoost | {'learning_rate': 0.5, 'n_estimators': 200} |
| LightGBM | {'learning_rate': 0.2, 'n_estimators': 200, 'num_leaves': 50} |
| Decision Tree | {'max_depth': None, 'min_samples_split': 2} |
| Gradient Boosting | {'learning_rate': 0.2, 'max_depth': 5, 'n_estimators': 300} |

**Supplementary Table S3 Hyper-parameter settings of the proposed model**
